# Phosphorus Dynamics in Managed and Natural Soils: SEM-PLS Analysis of *Vaccinium*, Forest, and Grassland Ecosystems

**DOI:** 10.3390/plants14020189

**Published:** 2025-01-11

**Authors:** Chun Lu, Soh Sugihara, Satoshi Noma, Haruo Tanaka, Ryosuke Tajima, Shingo Matsumoto, Dai Hirose, Xueyan Zhang, Ning Wang, Takuya Ban

**Affiliations:** 1 United Graduate School of Agricultural Science, Tokyo University of Agriculture and Technology, Tokyo 183-8509, Japan; chunlu2008@gmail.com (C.L.); noman@cc.tuat.ac.jp (S.N.); 2 Institute of Agriculture, Tokyo University of Agriculture and Technology, Tokyo 183-8509, Japan; sohs@cc.tuat.ac.jp (S.S.); haruo@cc.tuat.ac.jp (H.T.); 3 Graduate School of Agricultural Science, Tohoku University, Sendai 980-8572, Japan; 4 Graduate School of Natural Science and Technology, Shimane University, Shimane 690-8504, Japan; smatsu@life.shimane-u.ac.jp; 5 School of Pharmacy, Nihon University, Chiba 274-8555, Japan; hirose.dai@nihon-u.ac.jp; 6 College of Agriculture, Ningxia University, Yinchuan 750021, China; zhangxueyan123@sina.com; 7 Ningxia Baixin Agriculture Technology Development Co., Ltd., Yinchuan 750021, China; bxrlwn@163.com

**Keywords:** structural equation modeling (SEM), phosphorus fractionation, non-labile phosphorus (NLP), labile phosphorus (LP)

## Abstract

Phosphorus (P) availability in soils is often constrained by its accumulation in non-labile phosphorus (NLP) forms, limiting its accessibility to plants. This study examines how soil physical properties, chemical characteristics, and climatic conditions influence phosphorus fractionation and the transformation of NLP into plant-available labile phosphorus (LP). Utilizing global structural equation modeling (SEM), we found that silt content enhances organic phosphorus fractions, including NaHCO_3_-Po and NaOH-Po. In the upper 30 cm of soil, pH decreases the availability of NaHCO_3_-Po and NaOH-Po while stabilizing NLP, highlighting its essential role in phosphorus cycling under acidic conditions. In deeper soil layers, pH facilitates phosphorus mobilization from NLP pools, with effects varying across fractions. Long-term studies on Japanese *Vaccinium* soils reveal that pH and electrical conductivity (EC) management significantly promote NLP-to-LP conversion, primarily through NaOH-Po, thereby improving phosphorus use efficiency. These findings underscore the critical importance of prioritizing chemical property management over physical modifications to optimize nutrient cycling, preserve soil fertility, and reduce reliance on external phosphorus inputs in agricultural systems. Our study emphasizes the need for integrated approaches to achieve sustainable phosphorus management in both natural and managed ecosystems.

## 1. Introduction

Nitrogen (N) and phosphorus (P) are vital elements for terrestrial ecosystems, playing fundamental roles in plant growth, development, and the maintenance of soil health [1,2]. Unlike nitrogen, which is highly mobile in soil, phosphorus predominantly accumulates in non-labile forms, often creating a bottleneck for plant growth due to its limited bioavailability [3]. This accumulation is shaped by multiple factors, such as soil pH and microbial activity [4,5]. However, the dynamic interactions between N and P, particularly their relationship with soil phosphorus pools, remain challenging to monitor and understand [6,7].

As an essential macronutrient, phosphorus occurs in diverse forms within the soil, ranging from highly soluble to strongly recalcitrant, profoundly influencing nutrient cycling, microbial activity, and global soil health [8]. Although significant progress has been made in understanding phosphorus fractions in ecological and agricultural contexts [9,10], key questions remain about the biochemical pathways and environmental factors driving the transformation of non-labile phosphorus (NLP) into plant-available forms. This knowledge gap is particularly pronounced in crops like blueberries, which rely heavily on symbiotic relationships with mycorrhizal fungi to access phosphorus [11].

Species within the genus *Vaccinium*, such as blueberries, are shallow-rooted plants with fine roots that lack root hair, making them inefficient at directly absorbing phosphorus from the soil [12]. To overcome such limitations, these plants establish mutualistic relationships with ericoid mycorrhizal (ERM) fungi, which play a crucial role in mobilizing phosphorus from organic matter and non-labile pools in acidic soils [13]. This symbiosis is important for overcoming phosphorus limitations in nutrient-poor or acidic environments, directly contributing to plant productivity and ecosystem resilience [14]. Conversely, many herbaceous species and crops establish relationships with arbuscular mycorrhizal (AM) fungi that primarily facilitate the uptake of inorganic phosphorus (Pi) [15]. Together, AM and ERM fungi play complementary roles in phosphorus cycling, with AM fungi facilitating inorganic phosphorus uptake and ERM fungi enhancing the utilization of organic and non-labile phosphorus [16].

Although studies have explored the interactions among AM and ERM fungi, nitrogen and phosphorus dynamics, and phosphorus-solubilizing bacteria within the root zones of *Vaccinium* species [17], little attention has been given to phosphorus fractionation within these fungal-active layers. Specifically, the mechanisms controlling the conversion of non-labile phosphorus into bioavailable forms through phosphorus fractionation remain poorly understood. Our preliminary studies on Japanese blueberry fields revealed substantial accumulation of non-labile phosphorus within the 0–30 cm soil layer, a zone with the highest concentration of symbiotic fungal activity. The accumulation rates varied significantly among soil types, with phosphorus accumulation reaching 97% in Kuroboku soils (KS), 90% in Brown Forest soils (BFS), 55% in Red-Yellow soils (RYS), and 87% in Fluvic soils (FS) [18]. These findings highlight the necessity of understanding long-term phosphorus dynamics in fungal-active soils, offering critical insights into nutrient availability and guiding sustainable phosphorus management practices for *Vaccinium* plants like blueberries.

Phosphorus availability, a cornerstone of soil fertility and sustainable agriculture, is influenced not only by microbial and plant–microbe interactions, but also by environmental variables such as soil pH, temperature, and precipitation [19]. In soils, a significant portion of phosphorus is bound in recalcitrant forms, such as mineral-bound phosphorus and organic phosphorus, limiting its accessibility to plants [20]. Microorganisms, particularly ERM and AM fungi, play a crucial role in mobilizing these recalcitrant forms, converting them into soluble phosphorus that plants can absorb [21]. However, the precise roles of phosphorus fractions in regulating the transition from non-labile to labile forms in symbiotic zones remain unclear.

**Figure 1:**
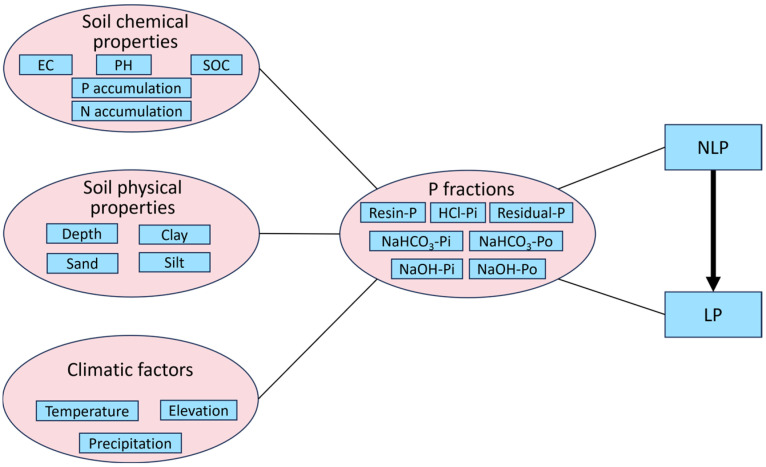
Predictive model for converting NLP to LP using phosphorus fractionation indices in connection with soil chemical and physical properties, as well as climatic factors.

In this study, we propose that specific phosphorus fractions are strongly associated with nitrogen and phosphorus accumulation in the soil and that extended *Vaccinium* cultivation modifies the relationship between these fractions and nutrient dynamics. Figure 1 illustrates the predictive model developed in this study to examine the transformation of non-labile phosphorus (NLP) into its labile form (LP). The model integrates phosphorus fractions (such as Residual-P, NaHCO_3_-Po, NaHCO_3_-Pi, NaOH-Po, NaOH-Pi, HCl-Pi, Resin-P) with soil chemical properties (such as pH, EC, SOC), soil physical properties (such as depth, sand, silt, clay), and climatic factors (such as temperature and precipitation). By incorporating these variables, we aim to elucidate how these factors collectively influence phosphorus transformations in long-term-cultivated *Vaccinium* soils.

To test our hypothesis, we constructed a conceptual structural equation model (SEM) based on global natural soil conditions. This model was then applied to comparative analyses of soils from long-term blueberry fields to assess the impact of sustained fertilization on phosphorus fractionation and accumulation. The SEM-PLS (structural equation modeling–partial least squares) method has been extensively applied in soil science to analyze intricate interactions among various factors [22]. Numerous studies have demonstrated the utility of SEM for predicting soil ecological dynamics and nutrient transformations [23,24,25]. By utilizing SEM-PLS models, we aim to forecast soil phosphorus dynamics, focusing on the long-term accumulation of nitrogen and phosphorus within blueberry cultivation areas.

**Figure 2:**
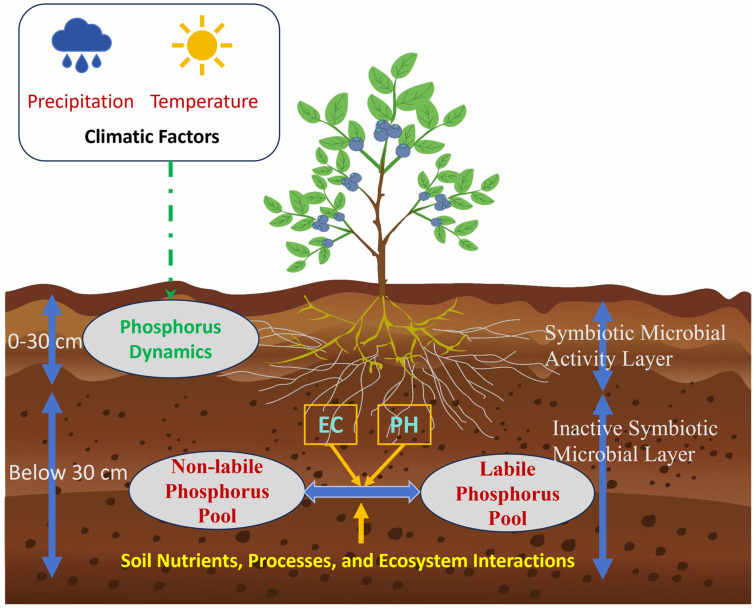
Soil profiles supporting the context of this study.

This study aims to validate the potential for transforming non-labile phosphorus into labile forms, providing a theoretical foundation for optimizing low-phosphorus fertilizer strategies and improving phosphorus use efficiency in *Vaccinium* soils. Our research will provide a robust framework for improving phosphorus management strategies in soils subjected to long-term *Vaccinium* cultivation, offering new insights into the pathways and processes governing phosphorus pool conversions. Figure 2 provides a visual representation of the soil layers in the study area, which forms the basis for understanding the phosphorus dynamics examined in this study.

## 2. Materials and Methods

### 2.1. Global Data Collection and Processing

The first dataset utilized in this study was derived from the global soil phosphorus distribution database compiled by He, Xianjin, et al. [26], which includes information on P fractions, pH, soil organic carbon (SOC), temperature, and precipitation. The initial dataset comprised 1857 records, but a series of preprocessing steps were undertaken to ensure data accuracy and completeness.

Initially, 171 records lacking soil depth information were removed to ensure that the subsequent analyses accurately reflected soil characteristics at specific depths. This resulted in a refined dataset referred to as Dataset 1 (see Supplementary File S1: global raw data.csv). In the second round of data cleaning, records without phosphorus fractionation data or geographic coordinates were excluded, as these variables were essential for the geospatial and soil nutrient analyses. The exclusion of these records produced Dataset 2 (see Supplementary File S2: global training data.csv).

**Figure 3:**
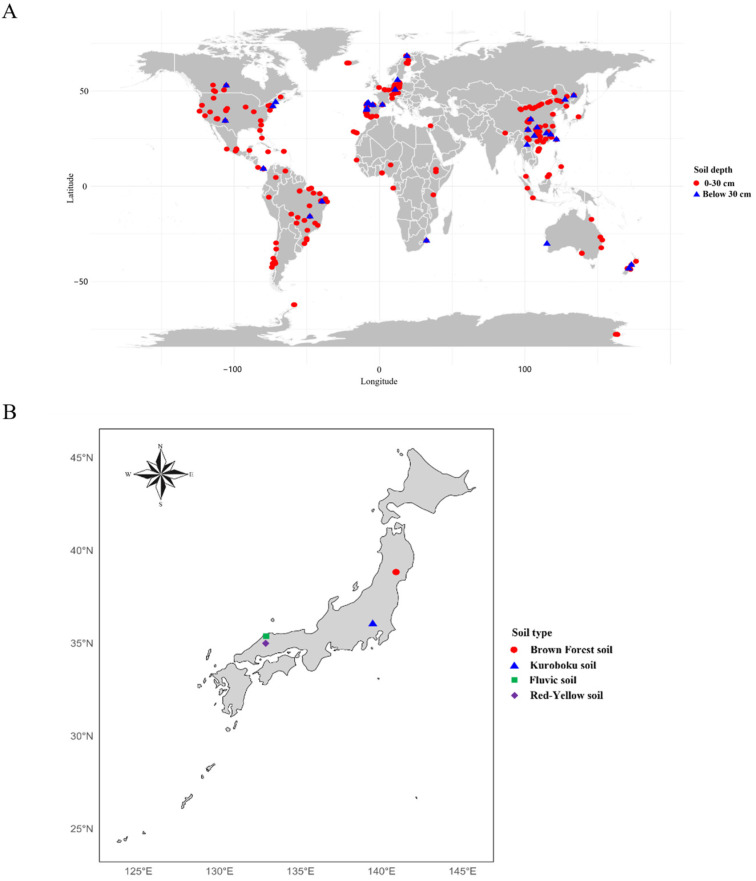
Global soil data collection sites (**A**) and a schematic diagram of soil data collection sites in Japan (**B**).

To address missing data in the remaining dataset, we utilized the MICE (Multiple Imputation by Chained Equations) approach implemented through the mice package in the R programming environment [27], with Predictive Mean Matching (PMM) applied for imputation. This process generated a complete dataset consisting of 676 records (Dataset 3, see Supplementary File S3: global predicted data.csv), the global distribution of which is illustrated in Figure 3A.

The dataset was then stratified by soil depth into two groups: 0–30 cm and below 30 cm. Each group was analyzed separately to assess the connections between soil properties and environmental factors, with related biome and soil information summarized in Table 1. The SEM model indicators for each group demonstrated good fit, further confirming the robustness of the models and their suitability for examining the relationships between soil properties, environmental factors, and nutrient dynamics across different soils.

The SEM (structural equation modeling) method was selected to analyze intricate interactions among various soil properties and phosphorus fractions across diverse ecosystems. Data cleaning procedures were rigorously applied to ensure the accuracy of global datasets, particularly for depth-specific analysis. Given the greater influence of soil chemical properties, such as pH, EC, and SOC, on phosphorus mobilization observed in global models, our analysis of Japanese blueberry soils focused exclusively on these variables, omitting soil texture and climatic factors which demonstrated weaker effects in global comparisons.

### 2.2. Soil Sampling and Processing in Japanese Blueberry Fields

Soil samples were obtained in 2022 from Japanese blueberry fields under continuous cultivation for over 20 years, encompassing four distinct soil types: Brown Forest soils (BFS), Kuroboku soils (KS), Red-Yellow soils (RYS), and Fluvic soils (FS), as shown in Figure 3B. Based on the World Reference Base for Soil Resources (WRB) [28], these soil types are classified as Cambisols (BFS), Andosols (KS), Acrisols (RYS), and Fluvisols (FS). For each soil type, samples were collected at depths of 30 cm and 60 cm from around five randomly selected blueberry trees. At each sampling location, a minimum of 500 g of soil was collected, then air-dried in a glasshouse, passed through a 2 mm sieve, and finely ground. A total of 120 samples were stored at 10 °C for later analysis.

Phosphorus fractionation was performed following the Hedley method [29], with modifications introduced by Tiessen and Moir [30], using a sequential extraction procedure. To determine Resin-P, 0.5 g of soil sample was mixed with 30 mL of deionized water and two anion exchange resin bags (AEM) and shaken at 25 °C for 16 h. The AEM bags were then removed, placed in 20 mL of 0.5 M HCl, and shaken for 2 h at 25 °C to release Resin-P, which was quantified using the molybdenum blue colorimetric method at 712 nm. For NaHCO_3_-extractable phosphorus, the soil residue was centrifuged at 3300 rpm for 20 min, and the supernatant discarded. The soil was then extracted with 30 mL of 0.5 M NaHCO_3_, shaken for 16 h at 25 °C, and centrifuged. The supernatant was analyzed for NaHCO_3_-Pi after dilution with distilled water and acidification with 0.9 M H_2_SO_4_, followed by colorimetric analysis at 712 nm. NaHCO_3_-Pt was determined by digesting another aliquot of the supernatant with ammonium persulfate at 120 °C for 1 h, with NaHCO_3_-Po calculated as the difference between NaHCO_3_-Pt and NaHCO_3_-Pi. Subsequently, the soil residue was extracted with 30 mL of 0.1 M NaOH under similar conditions. NaOH-Pi was measured after acidification with 0.9 M H_2_SO_4_, and NaOH-Pt was determined after digestion with ammonium persulfate at 120 °C for 90 min, with NaOH-Po calculated as the difference between NaOH-Pt and NaOH-Pi. The remaining soil was centrifuged, extracted with 20 mL of 1 M HCl for 16 h at 25 °C, and the supernatant analyzed for HCl-Pi. The phosphorus remaining in the soil after these steps was considered Residual-P, calculated by subtracting the sum of Resin-P, NaHCO_3_-P (Pi and Po), NaOH-P (Pi and Po), and HCl-Pi from the total phosphorus content. All phosphorus fractions were quantified using the molybdenum blue method at 712 nm.

Soil pH and electrical conductivity (EC) were determined using specific methods and varying soil-to-water ratios. For pH, a 1:2.5 ratio of soil to distilled water was used, with measurements taken via a HORIBA glass electrode (D-210P/220P, HORIBA Ltd., Kyoto, Japan). EC was measured using the AC bipolar method at a 1:5 soil-to-water ratio, utilizing a HORIBA device (D-210C/220C, HORIBA Ltd., Kyoto, Japan). The soil suspensions were shaken for 2 h, allowed to equilibrate, and the supernatant was subsequently analyzed for pH and EC.

The concentrations of soil organic carbon (SOC) and total nitrogen (N) were measured through dry combustion, employing a SUMIGRAPH NC-TR22 NC analyzer provided by Sumika Chemical Analysis Service, Ltd. (Osaka, Japan).

The total phosphorus content was determined through the HNO₃-HClO_4_ digestion technique [31]. Soil samples were combined with boiling stones and 10 mL of HNO₃, then heated at 200 °C. In the next step, HClO_4_ was introduced, and the heating process was maintained. Phosphorus levels were subsequently quantified via the molybdenum blue colorimetric method at a wavelength of 712 nm, using a SHIMADZU UV-1280 spectrophotometer (Shimadzu Corporation, Kyoto, Japan).

Nitrogen and phosphorus accumulation in Japanese blueberry fields was assessed by comparing total nitrogen and phosphorus concentrations in fertilized versus unfertilized soils.

### 2.3. Identification of AM and ERM Fungal Layers in Vaccinium Soils

The global distribution of *Vaccinium* species and their associated fungal layers was analyzed using data from the GBIF database [32]. The distribution records were downloaded and categorized based on the symbiotic association with arbuscular mycorrhizal (AM) or ericoid mycorrhizal (ERM) fungi.

**Figure 4:**
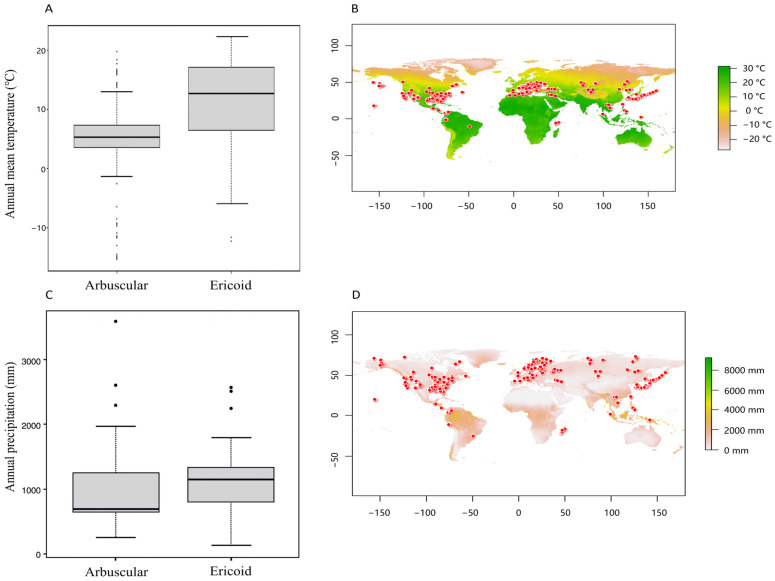
Comparison of temperature (**A**) and precipitation (**C**) in symbiotic environments of Arbuscular and Ericoid fungi associated with *Vaccinium* plants alongside their global distribution (**B**,**D**).

Problematic records were processed and corrected with the CoordinateCleaner package in the R system [33], and environmental data were obtained from WorldClim using the Raster package [34]. The climatic data, such as temperature and precipitation, were parsed and visualized; the global distribution of *Vaccinium* species is shown in Figure 4B,D.

We then filtered the data to identify the 0–30 cm soil layers where fungal activity was most pronounced based on the temperature and precipitation conditions associated with active symbiotic zones, as shown in Figure 4A,C. These filters helped distinguish between fungal-active and inactive soil layers, which were then used for further analysis.

### 2.4. Statistical Analysis

All data visualization, including graphs and tables, was carried out using R-4.4.0 software, PowerPoint 365, and Excel 365. Redundancy analysis (RDA) was conducted with Canoco version 5.0, while structural equation modeling (SEM) was implemented using Smart PLS 4.0. The SEM model fit indices, focusing on phosphorus fractions as key factors related to soil and environmental data, are shown in Table 2 [35].

## 3. Results

### 3.1. SEM Models for N and P Accumulation and Phosphorus Fractionation in Global Soils (0–30 cm and Below 30 cm)

**Figure 5:**
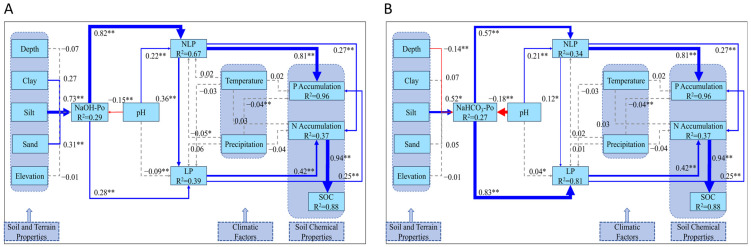
SEM model of the relationships among various phosphorus fractions within the upper 30 cm of soil and their interactions with soil and terrain properties, soil chemical properties, and climatic factors on a global scale. (**A**) The core factor in phosphorus fractions with NaOH-Po as the central component. (**B**) The core factor in phosphorus fractions with NaHCO_3_-Po as the central component. Note: Blue arrows represent positive correlations, while red arrows signify negative ones. Statistical significance is indicated by * for *p* < 0.05 and ** for *p* < 0.01. Solid lines correspond to statistically significant associations, whereas dashed lines represent non-significant interactions. R^2^ values indicate the explained variance of the respective variables.

**Figure 6:**
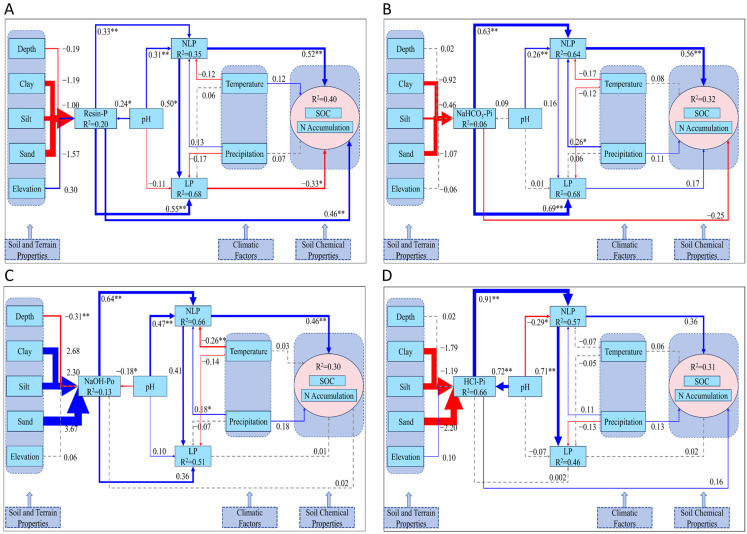
SEM model of the interactions among various phosphorus fractions within soil layers deeper than 30 cm and their associations with soil and terrain properties, soil chemical properties, and climatic factors on a global scale. (**A**) The core factor in phosphorus fractions with Resin-P as the central component. (**B**) The core factor in phosphorus fractions with NaHCO_3_-Pi as the central component. (**C**) The core factor in phosphorus fractions with NaOH-Po as the central component. (**D**) The core factor in phosphorus fractions with HCl-Pi as the central component. Note: Blue arrows represent positive correlations, while red arrows signify negative ones. Statistical significance is indicated by * for *p* < 0.05 and ** for *p* < 0.01. Solid lines correspond to statistically significant associations, whereas dashed lines represent non-significant interactions. R^2^ values indicate the explained variance of the respective variables.

The structural equation models (SEM) in Figure 5 and Figure 6 illustrate the complex interactions between phosphorus fractions, soil properties, and environmental factors in global soils at different depths. Within the upper 30 cm of soil (Figure 5), silt content is recognized as a significant factor that improves the availability of organic phosphorus fractions, such as NaOH-Po (R^2^ = 0.29) and NaHCO_3_-Po (R^2^ = 0.27), indicating that finer soil particles enhance phosphorus retention by binding phosphorus to organic matter [36]. Additionally, soil pH plays a dual role in phosphorus dynamics. It shows a significant negative effect on organic phosphorus fractions, such as NaOH-Po (β = −0.15, *p* < 0.01) and NaHCO_3_-Po (β = −0.18, *p* < 0.01), indicating that acidic conditions promote the release of phosphorus from these non-labile pools. However, pH positively regulates non-labile phosphorus (NLP), as shown in Figure 5A (β = 0.22, *p* < 0.01) and Figure 5B (β = 0.21, *p* < 0.01), suggesting that higher pH enhances the accumulation of NLP. Precipitation also shows a minor but statistically significant negative impact on NLP (β = −0.05, *p* < 0.05) and P accumulation (β = −0.04, *p* < 0.01). Moreover, the SEM highlights soil organic carbon’s (SOC, R^2^ = 0.88) significance in facilitating N accumulation (R^2^ = 0.37). Both LP and NLP act as critical intermediaries, directly influencing the accumulation of N and P in soils. This underscores the critical role of soil chemical properties and organic matter in maintaining N and P cycling within the upper soil layers, with LP and NLP linking phosphorus fractions to overall N and P availability.

In the deeper soil layer (below 30 cm, Figure 6), silt content has a positive effect on NaOH-Po, but negative effects on Resin-P, NaHCO_3_-Pi, and HCl-Pi, though none of these relationships are significant (*p* > 0.05), indicating a weaker influence compared to the upper soil layers. Soil pH exhibits significant positive effects on Resin-P and HCl-Pi, while its positive impact on NaHCO_3_-Pi is not significant. Conversely, pH shows a significant negative effect on NaOH-Po, highlighting its contrasting roles in phosphorus dynamics. Precipitation significantly enhances NLP in Figure 6B,C, while temperature in Figure 6C significantly reduces NLP. Overall, SOC and N accumulation are consistently positively and significantly correlated across Figure 6A–C, underscoring the critical role of soil organic carbon in driving nitrogen accumulation at this depth. However, in Figure 6A, LP, represented by Resin-P, shows a significant negative effect on both SOC and N accumulation, suggesting that labile phosphorus, in the form of Resin-P, may inhibit nitrogen accumulation or compete with SOC in this particular model.

**Figure 7:**
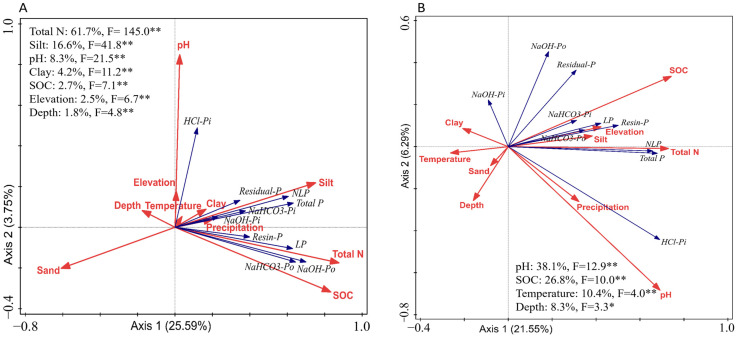
RDA analysis of global phosphorus fractionation concerning climatic factors and soil properties in the upper 30 cm (**A**) and deeper layers below 30 cm (**B**). Note: Asterisks (*) denote statistical significance, with * *p* ≤ 0.05 and ** *p* ≤ 0.01.

Figure 7 offers additional insights into the interactions among phosphorus fractions, soil properties, and climatic factors across two soil layers. Within the upper 30 cm of soil (Figure 7A), total N accounts for the largest proportion of phosphorus variation (61.7%), followed by silt content (16.6%), pH (8.3%), and clay content (4.2%). pH positively correlates with stable phosphorus forms like HCl-Pi, while it is negatively associated with organic fractions such as NaOH-Po and NaHCO_3_-Po. In the below 30 cm layer (Figure 7B), pH is the dominant factor (38.1%), followed by SOC (26.8%) and temperature (10.4%). At this depth, the influence of silt decreases, and phosphorus becomes increasingly stabilized in less labile and more stable forms like HCl-Pi, which are less readily available for plant uptake. Overall, Figure 7 emphasizes the pivotal influence of pH and SOC on phosphorus cycling, aligning with the insights presented in Figure 5 and Figure 6.

### 3.2. SEM Models for Nitrogen and Phosphorus Accumulation and Their Fractionation Across Various Biomes in Global Soils Within the Top 30 cm

**Figure 8:**
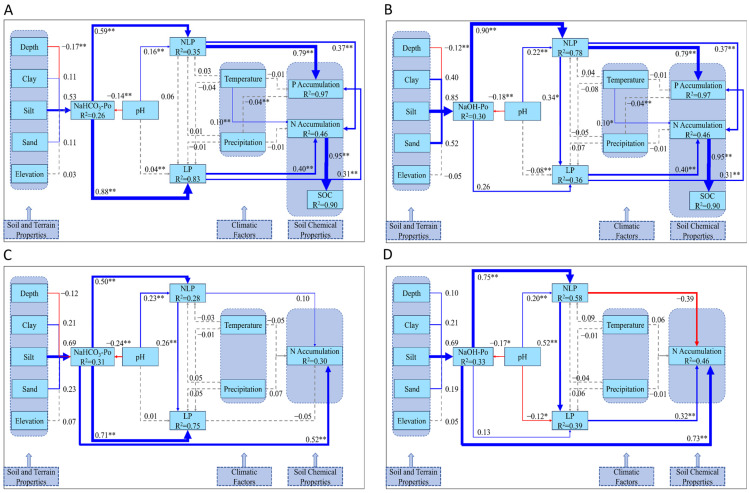
SEM models of the interactions among various phosphorus fractions within the upper 30 cm of soil and their relationships with soil and terrain properties, soil chemical properties, and climatic factors in forest soils (**A**,**B**) and grassland soils (**C**,**D**). Note: Blue arrows represent positive correlations, while red arrows signify negative ones. Statistical significance is indicated by * for *p* < 0.05 and ** for *p* < 0.01. Solid lines correspond to statistically significant associations, whereas dashed lines represent non-significant interactions. R^2^ values indicate the explained variance of the respective variables.

Comparative SEM analyses across forest and grassland biomes (Figure 8) reveal notable differences in phosphorus fractionation and nutrient accumulation driven by biome-specific soil properties. In forest soils (Figure 8A,B), although silt content had a positive impact on NaHCO_3_-Po and NaOH-Po, the effects lacked statistical significance (*p* > 0.05). Conversely, depth exhibited a markedly negative effect on both NaHCO_3_-Po (β = −0.17, *p* < 0.01) and NaOH-Po (β = −0.12, *p* < 0.01), highlighting the role of deeper soils in reducing phosphorus availability. Climatic factors, including temperature and precipitation, also played a role, with temperature exerting a significant positive effect on N accumulation, and precipitation showing a minor but measurable effect on phosphorus dynamics. NLP and LP positively influenced both P and N accumulation, with NLP having the most pronounced impact on P accumulation (β = 0.79, *p* < 0.01). The model also demonstrated that SOC (R^2^ = 0.90) played a central role in N accumulation but did not directly influence P accumulation, underscoring the importance of organic matter in regulating nitrogen cycling in forest ecosystems [37].

In contrast, grassland soils (Figure 8C,D) exhibited distinct phosphorus fractionation patterns. Silt content had a positive effect on NaHCO_3_-Po and NaOH-Po; however, the relationships did not reach statistical significance (*p* > 0.05). pH did not have a pronounced effect on LP, but it negatively influenced both NaHCO_3_-Po (β = −0.24, *p* < 0.01) and NaOH-Po (β = −0.17, *p* < 0.05), while positively affecting NLP (*p* < 0.01). Unlike forest soils, temperature and precipitation had weaker and non-significant effects on NLP, LP, and N accumulation in grasslands. Notably, only N accumulation was successfully modeled in grasslands, with NaHCO_3_-Po and NaOH-Po showing significant positive effects. Additionally, LP exhibited a strong positive impact on N accumulation in the NaOH-Po model.

**Figure 9:**
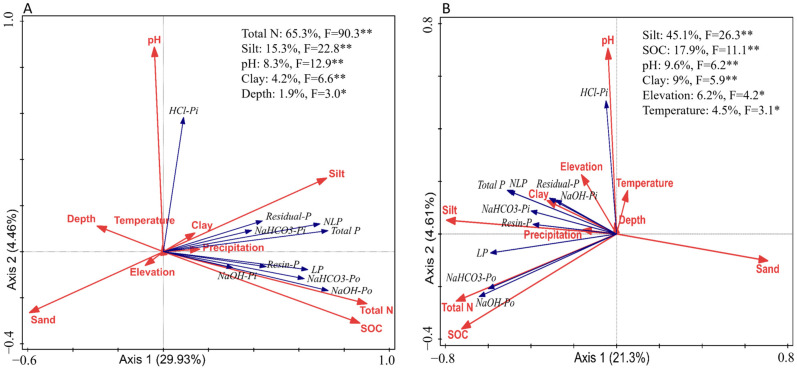
RDA analysis of global phosphorus fractionation concerning climatic factors and soil properties in forest soils (**A**) and grassland soils (**B**) within the 0–30 cm layer. Note: Asterisks (*) denote statistical significance, with * *p* ≤ 0.05 and ** *p* ≤ 0.01.

The redundancy analysis (RDA) in Figure 9 further confirms these biome-specific trends. In forest soils (Figure 9A), total nitrogen (65.3%) and silt content (15.3%) were the primary contributors to phosphorus distribution, with pH (8.3%) and clay (4.2%) also playing significant roles. The positive correlation between pH and HCl-Pi suggests that soil acidity enhances phosphorus retention in mineral-bound forms. In grasslands (Figure 9B), silt content (45.1%) and SOC (17.9%) were the dominant factors, with significant contributions from pH (9.6%) and clay content (9.0%). SOC’s positive association with NaOH-Po and NaHCO_3_-Po highlights the critical role of organic matter in phosphorus cycling regulation within grassland soils.

### 3.3. SEM Models for N and P Accumulation and Phosphorus Fractionation in Japanese Blueberry Soils at Depths of 0–30 cm and 30–60 cm

**Figure 10:**
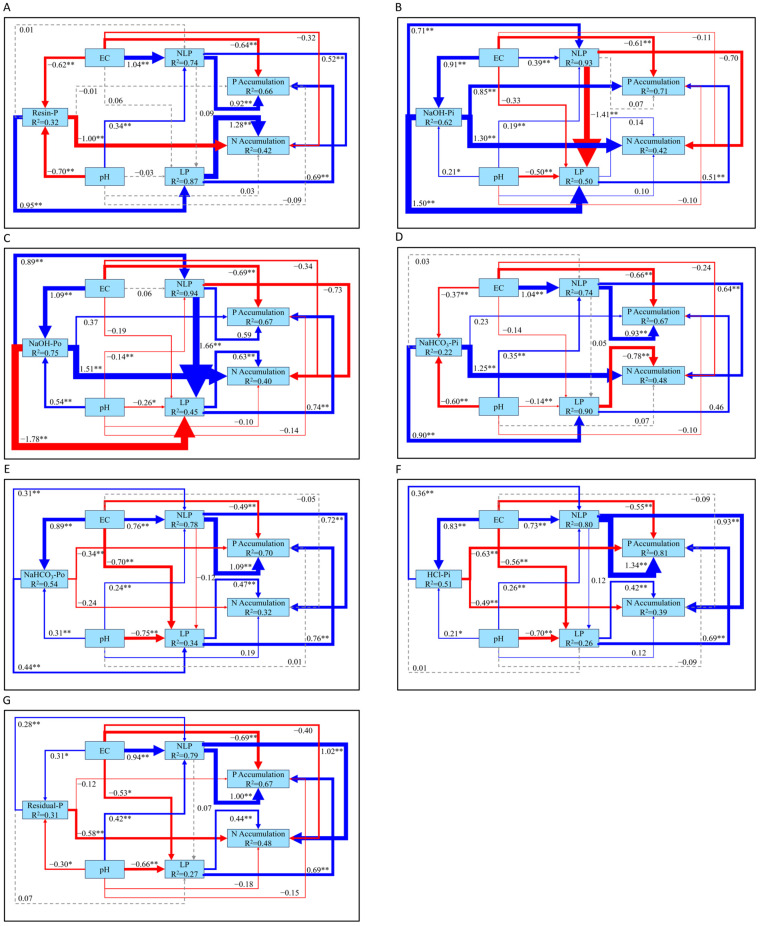
SEM model illustrating the interactions among various phosphorus fractions and soil properties within the upper 30 cm of soil in Japanese blueberry fields. (**A**) The core factor in phosphorus fractions with Resin-P as the central component. (**B**) The core factor in phosphorus fractions with NaOH-Pi as the central component. (**C**) The core factor in phosphorus fractions with NaOH-Po as the central component. (**D**) The core factor in phosphorus fractions with NaHCO_3_-Pi as the central component. (**E**) The core factor in phosphorus fractions with NaHCO_3_-Po as the central component. (**F**) The core factor in phosphorus fractions with HCl-Pi as the central component. (**G**) The core factor in phosphorus fractions with Residual-P as the central component. Note: Figure 10C,E are adapted from previously published work by Lu et al., 2024 [20]. Blue arrows represent positive correlations, while red arrows signify negative ones. Statistical significance is indicated by * for *p* < 0.05 and ** for *p* < 0.01. Solid lines correspond to statistically significant associations, whereas dashed lines represent non-significant interactions. R^2^ values indicate the explained variance of the respective variables.

**Figure 11:**
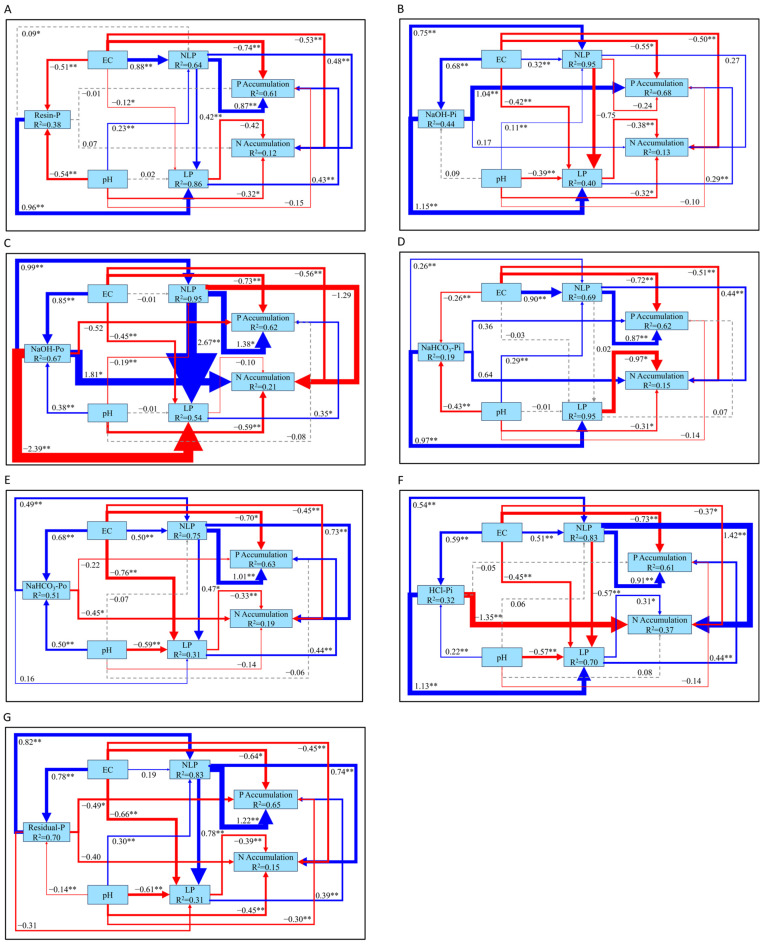
SEM model depicting the interactions among various phosphorus fractions and soil properties within the 30–60 cm soil layer in Japanese blueberry fields. (**A**) The core factor in phosphorus fractions with Resin-P as the central component. (**B**) The core factor in phosphorus fractions with NaOH-Pi as the central component. (**C**) The core factor in phosphorus fractions with NaOH-Po as the central component. (**D**) The core factor in phosphorus fractions with NaHCO_3_-Pi as the central component. (**E**) The core factor in phosphorus fractions with NaHCO_3_-Po as the central component. (**F**) The core factor in phosphorus fractions with HCl-Pi as the central component. (**G**) The core factor in phosphorus fractions with Residual-P as the central component. Note: Figure 11C,E is adapted from previously published work by Lu et al., 2024 [20]. Blue arrows represent positive correlations, while red arrows signify negative ones. Statistical significance is indicated by * for *p* < 0.05 and ** for *p* < 0.01. Solid lines correspond to statistically significant associations, whereas dashed lines represent non-significant interactions. R^2^ values indicate the explained variance of the respective variables.

**Figure 12:**
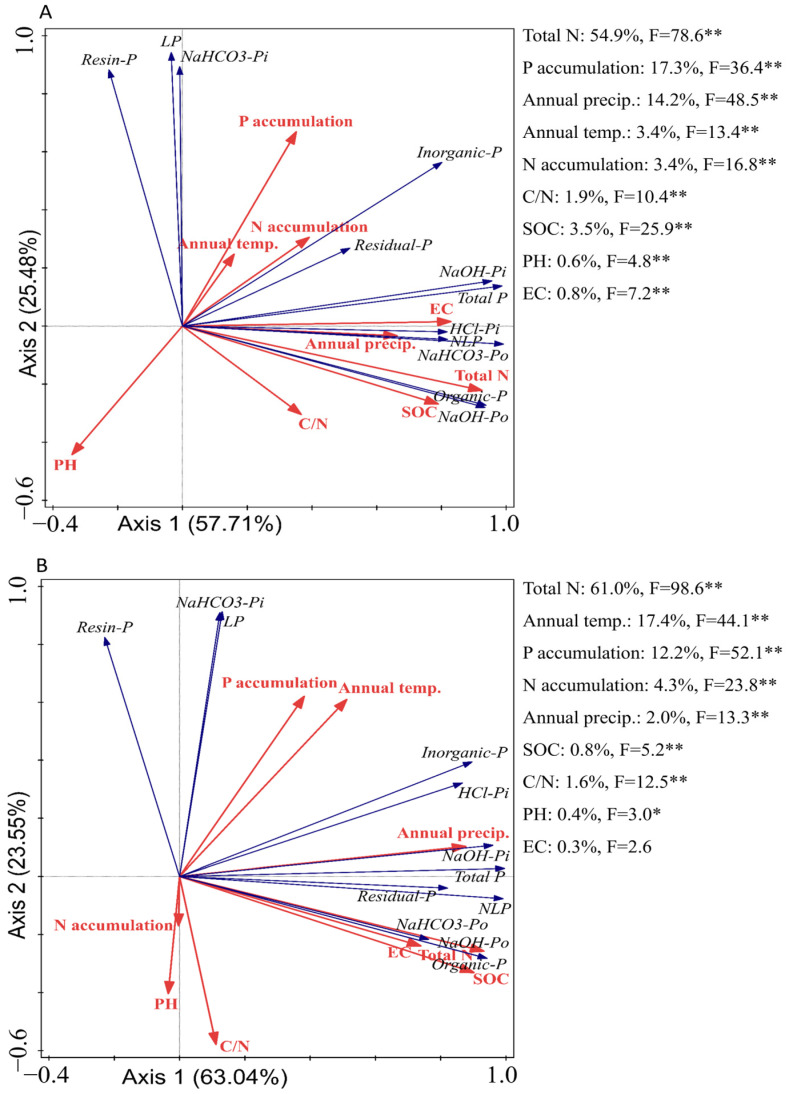
RDA analysis of Japanese blueberry field’s phosphorus fractionation concerning climatic factors and soil properties in 0–30 cm soil layer (**A**) and 30–60 cm soil layer (**B**). Note: Annual temp. refers to Annual Temperature, while Annual precip. refers to annual precipitation. The temperature and precipitation data were sourced from the Japan Meteorological Agency [38]. Asterisks (*) denote statistical significance, with * *p* ≤ 0.05 and ** *p* ≤ 0.01.

Within Japanese blueberry soils, the SEM models (Figure 10 and Figure 11) elucidate the intricate dynamics between phosphorus fractions and soil attributes across multiple depths. In the upper 30 cm of soil (Figure 10), pH showed contrasting effects on phosphorus fractions, negatively influencing Resin-P (β = −0.70, *p* < 0.01), NaHCO_3_-Pi (β = −0.60, *p* < 0.01), and Residual-P (β = −0.30, *p* < 0.05), while positively affecting more stable forms like NaOH-Po (β = 0.54, *p* < 0.01), NaHCO_3_-Po (β = 0.31, *p* < 0.01), and HCl-Pi (β = 0.21, *p* < 0.05). pH exhibited a strong positive influence on NLP in most models, except for a negative effect in Figure 10C, and negatively impacted LP in several models (Figure 10B–G). Redundancy analysis (Figure 12) similarly revealed that pH had a consistently negative association with labile phosphorus fractions, including Resin-P and NaHCO_3_-Pi, across both soil layers. This suggests that higher pH levels reduce P availability by promoting the formation of insoluble phosphorus compounds, further corroborating the SEM findings.

EC had negative effects on Resin-P (β = −0.62, *p* < 0.01) and NaHCO_3_-Pi (β = −0.37, *p* < 0.01) but promoted stable phosphorus forms, such as NaOH-Po (β = 1.09, *p* < 0.01), NaOH-Pi (β = 0.91, *p* < 0.01), HCl-Pi (β = 0.83, *p* < 0.01), and Residual-P (β = 0.31, *p* < 0.05). These findings align with the RDA results (Figure 12), which highlighted a weaker but notable influence of EC, particularly in the deeper soil layer, where its indirect role in modulating phosphorus cycling becomes more pronounced. NLP positively influenced both N and P accumulation in several models, while LP showed positive effects on N and P accumulation, except in Figure 10D, where it had a negative effect on N accumulation.

As we move to the 30–60 cm layer of soil (Figure 11), similar patterns persist, but certain effects become more pronounced. pH continued to negatively affect Resin-P (β = −0.54, *p* < 0.01), NaHCO_3_-Pi (β = −0.43, *p* < 0.01), and Residual-P (β = −0.14, *p* < 0.01), while positively influencing NaOH-Po (β = 0.38, *p* < 0.01), NaHCO_3_-Po (β = 0.50, *p* < 0.01), and HCl-Pi (β = 0.22, *p* < 0.01). pH significantly promoted NLP in most models, except for a negative effect in Figure 11C, and it had a negative impact on LP in multiple models. EC followed a similar trend as in the upper layer, negatively affecting labile phosphorus fractions but promoting stable forms like NaOH-Po (β = 0.85, *p* < 0.01), NaOH-Pi (β = 0.68, *p* < 0.01), HCl-Pi (β = 0.59, *p* < 0.01), and Residual-P (β = 0.78, *p* < 0.01). NLP positively influenced both N and P accumulation, though in Figure 11C, it negatively impacted N accumulation without significance. LP typically exhibited a negative impact on N accumulation but contributed positively to P accumulation, with Figure 11F showing positive effects on both. Climatic factors also emerged as key drivers of phosphorus dynamics (Figure 12). In the surface layer, annual precipitation was identified as a dominant factor influencing P fractions, while, in the deeper layer, annual temperature played a more significant role. These results underscore the interplay between soil properties and environmental drivers in shaping depth-dependent phosphorus availability.

Moreover, in the global SEM model, soil pH and soil organic carbon (SOC) emerged as the primary drivers of phosphorus fractionation, playing a pivotal role in converting NLP to LP. These results are consistent with those observed in the Japanese blueberry soils, where pH and EC played dominant roles in phosphorus dynamics. However, the more localized analysis revealed a complex interplay in the Japanese blueberry soils, where pH and EC regulated the transformation of NLP to LP through specific phosphorus fractions. In the 0–30 cm soil layer, NaOH-Po emerged as the key fraction driving this transformation, while, in the 30–60 cm soil layer, the critical fractions included Resin-P, NaOH-Po, NaHCO_3_-Po, and Residual-P.

### 3.4. SEM Models Addressing Nitrogen and Phosphorus Accumulation and Phosphorus Fractionation in Active and Inactive Symbiotic Fungal Layers

To enhance our understanding of the interaction between *Vaccinium* plants and their associated symbiotic fungi, we first analyzed the environmental requirements (temperature and precipitation) for arbuscular mycorrhizal (AM) and ericoid mycorrhizal (ERM) fungi symbiosis (Figure 4). Figure 4 compares temperature (Figure 4A) and precipitation (Figure 4C) in symbiotic environments for AM and ERM fungi associated with *Vaccinium* plants, alongside their global distribution (Figure 4B,D). The temperature for AM fungi shows a median of approximately 5 °C, ranging from −10 °C to 15 °C, while ERM fungi exhibits a higher median temperature of 14 °C with a broader range of −5 °C to over 25 °C. Precipitation shows a median value of 1000 mm for AM fungi and 1100 mm for ERM fungi, indicating that ERM fungi prefer slightly wetter environments.

**Figure 13:**
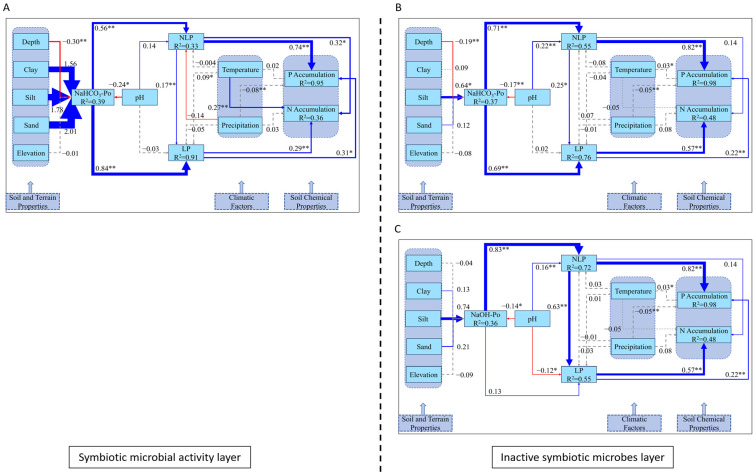
SEM models illustrate the interactions among various phosphorus fractions within the symbiotic microbial activity layer (**A**) and the inactive symbiotic microbial layers (**B**,**C**). Note: Blue arrows represent positive correlations, while red arrows signify negative ones. Statistical significance is indicated by * for *p* < 0.05 and ** for *p* < 0.01. Solid lines correspond to statistically significant associations, whereas dashed lines represent non-significant interactions. R^2^ values indicate the explained variance of the respective variables.

Using these findings, we filtered phosphorus fractionation data from the upper 30 cm of soil (the rhizosphere for *Vaccinium* plants and their symbiotic fungi) across global soils, focusing on areas within the optimal ranges for AM and ERM symbiosis (temperature: 5–14 °C, precipitation: 800–1500 mm). This allowed us to model the phosphorus dynamics in symbiotic active and inactive layers (Figure 13).

In the symbiotic microbial activity layer (Figure 13A), silt had a positive but non-significant effect on NaHCO_3_-Po (R^2^ = 0.39), while depth significantly negatively influenced NaHCO_3_-Po. pH also showed a negative effect on NaHCO_3_-Po (β = −0.24, *p* < 0.05). NaHCO_3_-Po positively influenced NLP and LP, subsequently promoting the accumulation of both P (R^2^ = 0.95) and N (R^2^ = 0.36). Temperature had a significant positive impact on N accumulation. In contrast, in the inactive microbial layers (Figure 13B,C), silt significantly influenced NaHCO_3_-Po (R^2^ = 0.37), but soil properties did not significantly impact NaOH-Po (R^2^ = 0.36). pH negatively influenced both NaHCO_3_-Po and NaOH-Po. Similar to the active layer, NLP and LP continued to drive the accumulation of P and N, though temperature and precipitation played a lesser role.

### 3.5. Summary of Key Findings from SEM Models

Due to the complexity and abundance of the SEM structural models presented in this study, we have provided a concise summary to enhance reader comprehension. The summary of key findings from SEM models is presented in Table 3, offering a clear overview of the main relationships and insights derived from the analyses.

## 4. Discussion

### 4.1. SEM Models for N and P Accumulation and Phosphorus Fractionation in Global Soils (0–30 cm and Below 30 cm)

The SEM models (Figure 5) clearly demonstrate the critical roles of soil texture, particularly silt content, pH, and SOC, in regulating P fractions and accumulation within the upper 30 cm of soil. As indicated by the results, silt content has a positive influence on NaOH-Po and NaHCO_3_-Po, while pH negatively affects these phosphorus fractions, showing that the interaction between soil texture and pH is crucial for P mobilization. A key process observed here is the significant transformation of NLP into LP through the negative regulation of pH on NaOH-Po and NaHCO_3_-Po, suggesting that decreasing pH enhances this conversion. This underscores the role of acidic conditions in promoting the transformation of less accessible phosphorus into more labile forms [39].

In addition to P accumulation, N accumulation is also significant in the upper soil layers, a zone where nutrient cycling and plant growth are particularly active [40]. Figure 5 illustrates that SOC not only promotes P accumulation, but also has a strong positive effect on N accumulation, consistent with previous studies showing that organic matter enhances both N and P availability by releasing nutrients during decomposition and promoting microbial activity [41].

In contrast, the dynamics shift in deeper soils (below 30 cm, Figure 6), where pH exhibits a positive relationship with the conversion of NLP to LP. Specifically, higher pH promotes the conversion of NLP, particularly Resin-P and HCl-Pi, into LP, suggesting that alkaline conditions in deeper layers facilitate phosphorus mobilization into more bioavailable forms. This finding is in opposition to the upper soil layers, where acidic conditions prevail in driving phosphorus transformation, suggesting a depth-related contrast in how pH modulates phosphorus forms [42]. Precipitation, while having a significant effect in the upper layers, shows less influence below 30 cm, where N accumulation is increasingly controlled by SOC, implying that deeper soils rely more on intrinsic soil properties rather than climatic factors [43].

The redundancy analysis (RDA) in Figure 7 further supports these insights. Within the upper 30 cm of soil, total N, pH, and silt content are the main drivers of N and P accumulation, while, in deeper soils, SOC plays a more dominant role. These findings underscore the increasing significance of organic matter in deeper soil layers, where lower biological activity and slower decomposition rates position SOC as a crucial driver of nutrient dynamics, especially concerning nitrogen [44].

### 4.2. SEM Models for Nitrogen and Phosphorus Accumulation and Their Fractionation Across Various Biomes in Global Soils Within the Top 30 cm

The SEM models (Figure 8) reveal distinct patterns of nitrogen and phosphorus accumulation in forest and grassland soils. In forest soils (Figure 8B), the negative regulation of NaOH-Po by pH suggests that acidic conditions favor the conversion of NLP into LP, thereby enhancing phosphorus availability. This finding aligns with the well-documented role of forest soil acidity in driving nutrient mobilization, where the conversion of more stable phosphorus forms to labile phosphorus supports plant nutrient uptake [45,46].

In grassland soils (Figure 8C,D), a similar process occurs, with pH negatively regulating both NaHCO_3_-Po and NaOH-Po, indicating that the conversion of NLP to LP is also pH-dependent in these ecosystems. Unlike forest soils (Figure 8A,B), SOC does not appear in the SEM pathways involving these phosphorus fractions; yet, grassland soils exhibit a more efficient phosphorus mobilization, driven by the stronger pH-mediated regulation of NaHCO_3_-Po and NaOH-Po. These observations indicate a biome-specific divergence in the interaction between soil properties and pH in controlling phosphorus mobilization [47,48].

However, in grassland soils, phosphorus cycling appears to be more strongly influenced by soil properties such as silt content and pH regulation of NaHCO_3_-Po and NaOH-Po, while SOC plays a significant but less dominant role compared to its influence in forest soils. This highlights a biome-specific variation, with grassland soils exhibiting a stronger dependency on soil texture and specific phosphorus fractions for nutrient mobilization.

The redundancy analysis (RDA) in Figure 9 further distinguishes these biome-specific trends. In forest soils (Figure 9A), total nitrogen and silt content play critical roles in phosphorus accumulation, while, in grasslands (Figure 9B), SOC and silt content dominate. Such divergence reinforces the idea that the mechanisms driving phosphorus fractionation and NLP conversion vary between ecosystems, likely due to differing interactions among pH, texture, and organic matter [49].

### 4.3. SEM Models for N and P Accumulation and Phosphorus Fractionation in Japanese Blueberry Soils at Depths of 0–30 cm and 30–60 cm

Within the context of global soils, soil physical properties such as silt, alongside chemical properties like pH, play critical roles in regulating phosphorus fractions, with the potential to influence the conversion of NLP into LP. Climatic factors contribute to these processes, although to a lesser extent. However, due to the inherent difficulty in altering silt content, our study focused on soil chemical properties—specifically pH and electrical conductivity (EC)—as key levers to drive phosphorus fractionation in Japanese *Vaccinium* soils.

SEM models across the 0–30 cm and 30–60 cm layers reveal that pH and EC are significant factors in modulating the conversion of NLP to LP, highlighting their potential to enhance phosphorus availability in these long-term-cultivated soils. Within the upper 30 cm of soil (Figure 10), pH and EC positively influence the conversion of NLP, especially NaOH-Po, into LP, indicating that acidic conditions promote the release of phosphorus from organic and non-labile pools into more labile forms, thus increasing phosphorus availability for plant uptake [50]. Interestingly, the regulation of NaOH-Pi by pH and EC displays a distinct pattern, where higher pH and EC increase NaOH-Pi levels but simultaneously inhibit the net transformation of NLP into LP. This pattern can be attributed to the dual role of NaOH-Pi as a transitional pool, which enhances both NLP and LP accumulation, thereby reducing the efficiency of NLP-to-LP conversion.

Redundancy analysis (Figure 12) further supports these observations, revealing that pH consistently exhibits a negative association with labile phosphorus fractions, such as Resin-P and NaHCO_3_-Pi, across both soil layers. This highlights the inhibitory effect of higher pH on the availability of bioavailable phosphorus, likely due to the precipitation of insoluble phosphorus compounds. EC, while exerting a weaker influence overall, displays a notable role in modulating stable phosphorus fractions such as Residual-P and NaOH-Po, particularly in the deeper soil layer (30–60 cm). These findings suggest that the interaction between pH, EC, and phosphorus fractions is both depth-dependent and fraction-specific, contributing to the complexity of phosphorus dynamics in long-term-cultivated soils.

In the 30–60 cm layer (Figure 11), the patterns remain similar, with pH and EC continuing to promote the conversion of NLP to LP through fractions such as NaOH-Po, NaHCO_3_-Po, Resin-P, and Residual-P. Notably, the effect of pH and EC on HCl-Pi shows a nuanced difference: higher pH and EC reduce the conversion of NLP to LP in this fraction. This observation aligns with the RDA findings (Figure 12), which reveal depth-specific variations in climatic factors, such as annual temperature in the deeper soil layer, further modulating phosphorus availability and fractionation. Such climatic effects may indirectly influence phosphorus dynamics by altering organic matter turnover and nutrient cycling, as indicated by the strong associations between SOC, C/N, and labile phosphorus fractions in the surface soil [51]. Altogether, these interactions highlight the complexity of phosphorus mobilization in deeper layers, where chemical properties, depth, and phosphorus fractions interact in more intricate ways [52,53].

Globally, while factors like silt and pH are known to drive phosphorus dynamics, the difficulty of altering soil texture in agricultural systems makes focusing on modifiable soil chemical properties more pragmatic [54,55]. In the Japanese blueberry fields studied, we observed that the targeted manipulation of pH and EC offers a feasible strategy for enhancing phosphorus use efficiency. By concentrating on these chemical properties, our study demonstrates the potential to effectively regulate phosphorus fractionation and promote the conversion of NLP to LP, providing valuable insights for improving phosphorus management in long-term-cultivated soils, particularly under acidic conditions typical of *Vaccinium* cultivation.

### 4.4. SEM Models Addressing Nitrogen and Phosphorus Accumulation and Phosphorus Fractionation in Active and Inactive Symbiotic Fungal Layers

In the active symbiotic microbial layer (Figure 13A), pH exerts a significant effect on NaHCO_3_-Po, promoting the conversion of NLP into LP. This underscores the essential role of microbial activity in facilitating phosphorus mobilization and cycling, where biological processes are actively driving the transformation of phosphorus into plant-available forms [56]. Temperature also plays a significant role in enhancing N accumulation, further reinforcing the importance of biological activity in these layers [57]. Interestingly, studies by Hugo A. Pantigoso et al. [58] demonstrated that phosphorus fertilization reduces the ability of phosphate-solubilizing microbes to convert NLP into LP. Although their study did not specifically address phosphorus fractionation, the SEM results from the active symbiotic layer suggest that, under biologically active conditions, phosphorus fractionation is dominated by microbial-driven pathways, with NaHCO_3_-Po as the primary contributor to the NLP-to-LP transformation. This observation aligns with the notion that microbial processes play a central role in regulating phosphorus pools in active layers.

In contrast, the inactive symbiotic microbial layers (Figure 13B,C) show that the pH regulation of NaHCO_3_-Po and NaOH-Po also promotes the conversion of NLP to LP, though the absence of active microbial processes means that these transformations rely more heavily on soil chemical properties. This indicates that, in the absence of microbial activity, pH and EC become the dominant factors in phosphorus cycling [59].

### 4.5. Implications for Long-Term Soil Management and Global Soil Health

The results of our global SEM analysis, supported by findings from long-term studies in Japanese blueberry fields, underscore the pivotal role of soil chemical properties—particularly pH and EC—in regulating phosphorus availability and cycling. By fostering environments that promote the efficient conversion of non-labile phosphorus into plant-accessible labile phosphorus, sustainable and low-input agricultural systems can be achieved [60,61]. In acidic soils, which are common in *Vaccinium* cultivation, targeted adjustments to pH and EC significantly enhance phosphorus bioavailability, reducing dependence on external phosphorus inputs while promoting efficient nutrient cycling. Furthermore, the ability to manipulate NaOH-Po and NaHCO_3_-Po pools through pH and EC adjustments represents a critical area for future research, with broad implications for nutrient management across diverse soils and ecosystems [62,63]. These findings provide actionable strategies for optimizing phosphorus dynamics and enhancing soil health, aligning with the principles of sustainable intensification and environmental stewardship.

Globally, soil health is shaped by the complex interplay of biological, chemical, and physical factors, with phosphorus availability serving as a cornerstone of nutrient dynamics and agricultural productivity [64]. Our findings demonstrate that depth-specific management strategies, informed by local soil properties, can optimize phosphorus mobilization and stabilization. For example, acidic conditions in surface soils facilitate the transformation of NLP into LP, supporting plant uptake and microbial activity, whereas alkaline conditions in deeper layers stabilize phosphorus in less-bioavailable forms. By targeting modifiable soil properties such as pH and EC, rather than less malleable factors like soil texture, these approaches offer scalable solutions for enhancing nutrient use efficiency and minimizing environmental impacts. Future research should refine these strategies across varied cropping systems and ecological contexts, advancing global soil health, food security, and the sustainability of agricultural systems.

## 5. Conclusions

This study clarifies the crucial role of soil chemical properties, particularly pH and EC, in driving the conversion of NLP into LP in long-term-cultivated *Vaccinium* soils. Our findings demonstrate that, although global phosphorus dynamics are shaped by soil physical characteristics such as silt and chemical factors like pH, the challenges of altering physical properties highlight the need to prioritize chemical management. In the acidic conditions of Japanese *Vaccinium* soils, targeted adjustments in pH and EC emerge as key strategies for improving phosphorus use efficiency. Structural equation modeling (SEM) reveals that, in the upper 30 cm of soil, acidic environments promote the conversion of NLP into plant-available forms, especially NaOH-Po, while higher EC promotes the transformation of NLP into bioavailable phosphorus fractions. In deeper soil layers, pH and EC interact in more complex ways depending on the specific phosphorus fractions and depth. Additionally, phosphorus and nitrogen accumulation are closely linked, with soil organic carbon (SOC) playing a significant role in nutrient cycling. By optimizing these modifiable chemical factors, particularly in acidic *Vaccinium* soils under long-term cultivation, phosphorus management can be potentially enhanced, leading to more sustainable and low-input systems for *Vaccinium* plants. Additionally, integrating chemical management with biological processes such as symbiotic fungal activity presents a promising approach for improving soil fertility and reducing external phosphorus inputs, thereby supporting sustainable cultivation practices.

## Figures and Tables

**Table 1:** Summary of literature data by soil depth and biomes.

Soil Depth	Biomes	Number of Literature Data	Number of References	Publication Year Range
0–30 cm	Desert	17	4	2007–2020
	Forest	280	93	1985–2021
	Grassland	169	38	1985–2021
	Permanent	16	5	1999–2011
	Savan	20	10	1992–2019
	Shrubland	37	16	1990–2020
	Tundra	18	4	2012–2017
	Wetland	33	8	2005–2018
Below 30 cm	Desert	1	1	2020
	Forest	51	18	1999–2021
	Grassland	20	9	1985–2020
	Savan	3	1	2011
	Shrubland	11	4	2004–2016

**Table 2:** Fit indices of models with P fractions as core factors in relation to soil and environmental data.

Figure Caption	The Core Factor in P Fractions	SRMR	d_ULS	d_G	Chi-Square	NFI	Notes
Figure 5A	NaOH-Po	0.07	0.53	0.18	485.03	0.93	Global model for the upper 30 cm of soil
Figure 5B	NaHCO_3_-Po	0.07	0.51	0.11	318.30	0.96	Global model for the upper 30 cm of soil
Figure 6A	Resin-P	0.08	0.57	0.35	151.53	0.86	Global model for soil layers beneath 30 cm depth
Figure 6B	NaHCO_3_-Pi	0.08	0.59	0.39	159.06	0.86	Global model for soil layers beneath 30 cm depth
Figure 6C	NaOH-Po	0.07	0.48	0.36	146.59	0.87	Global model for soil layers beneath 30 cm depth
Figure 6D	HCl-Pi	0.08	0.51	0.34	137.73	0.88	Global model for soil layers beneath 30 cm depth
Figure 8A	NaHCO_3_-Po	0.07	0.57	0.12	165.06	0.96	Global model of forest soil within the upper 30 cm layer
Figure 8B	NaOH-Po	0.07	0.50	0.21	258.60	0.93	Global model of forest soil within the upper 30 cm layer
Figure 8C	NaHCO_3_-Po	0.07	0.33	0.07	60.90	0.94	Global model of grassland soil within the upper 30 cm layer
Figure 8D	NaOH-Po	0.06	0.28	0.10	78.09	0.93	Global model of grassland soil within the upper 30 cm layer
Figure 10A	Resin-P	0.06	0.11	0.20	60.60	0.88	Model of Japan’s soil profile in the uppermost 30 cm
Figure 10B	NaOH-Pi	0.05	0.06	0.11	36.41	0.93	Model of Japan’s soil profile in the uppermost 30 cm
Figure 10C	NaOH-Po	0.06	0.09	0.15	48.10	0.92	Model of Japan’s soil profile in the uppermost 30 cm
Figure 10D	NaHCO_3_-Pi	0.06	0.09	0.17	53.88	0.90	Model of Japan’s soil profile in the uppermost 30 cm
Figure 10E	NaHCO_3_-Po	0.06	0.09	0.14	45.53	0.89	Model of Japan’s soil profile in the uppermost 30 cm
Figure 10F	HCl-Pi	0.04	0.05	0.11	38.22	0.91	Model of Japan’s soil profile in the uppermost 30 cm
Figure 10G	Residual-P	0.06	0.09	0.17	53.02	0.87	Model of Japan’s soil profile in the uppermost 30 cm
Figure 11A	Resin-P	0.07	0.12	0.10	34.33	0.91	Model of Japan’s soil profile at depths of 30–60 cm
Figure 11B	NaOH-Pi	0.06	0.11	0.12	40.77	0.91	Model of Japan’s soil profile at depths of 30–60 cm
Figure 11C	NaOH-Po	0.07	0.14	0.15	49.11	0.91	Model of Japan’s soil profile at depths of 30–60 cm
Figure 11D	NaHCO_3_-Pi	0.06	0.11	0.10	33.05	0.93	Model of Japan’s soil profile at depths of 30–60 cm
Figure 11E	NaHCO_3_-Po	0.06	0.10	0.09	31.30	0.90	Model of Japan’s soil profile at depths of 30–60 cm
Figure 11F	HCl-Pi	0.06	0.12	0.15	49.48	0.88	Model of Japan’s soil profile at depths of 30–60 cm
Figure 11G	Residual-P	0.06	0.10	0.09	32.38	0.92	Model of Japan’s soil profile at depths of 30–60 cm
Figure 13A	NaHCO_3_-Po	0.08	0.62	0.19	88.24	0.93	Global model of symbiotic microbial activity within the upper 30 cm of soil
Figure 13B	NaHCO_3_-Po	0.06	0.35	0.14	137.84	0.94	Global model of inactive symbiotic microbes within the upper 30 cm of soil
Figure 13C	NaOH-Po	0.06	0.34	0.21	178.55	0.92	Global model of inactive symbiotic microbes within the upper 30 cm of soil

**Table 3:** Summary of key findings from structural equation models.

Figure Caption	The Core Factor in P Fractions (Key Variables)	Path Relationships	Significance (*p*-Value)	Key Findings
Figure 5A	NaOH-Po (pH, NLP, Silt, LP)	pH positively affects NLP; silt positively affects NaOH-Po; NLP positively affects LP.	pH→NLP (*p* < 0.01). Silt→NaOH-Po (*p* < 0.01). NLP→LP (*p* < 0.01).	pH and silt content regulate phosphorus availability and retention in upper soils. Acidic conditions promote the release of phosphorus from these non-labile pools.
Figure 5B	NaHCO_3_-Po (pH, NLP, Silt, LP)	pH positively regulates NLP; silt positively affects NaHCO_3_-Po; NLP positively affects LP.	pH→NLP (*p* < 0.01). Silt→NaHCO_3_-Po (*p* < 0.05). NLP→LP (*p* < 0.05).	Higher pH enhances NLP. Precipitation reduces phosphorus accumulation.
Figure 6A	Resin-P (pH, NLP, LP, SOC, N Accumulation)	pH positively regulates NLP and Resin-P; Resin-P positively affects NLP, LP, SOC, and N accumulation; NLP positively affects LP.	pH→NLP (*p* < 0.01). pH→ Resin-P (*p* < 0.05). Resin-P→NLP (*p* < 0.01). Resin-P→LP (*p* < 0.01). Resin-P→SCO and N Accumulation (*p* < 0.01).	SOC and N accumulation are consistently positively and significantly correlated. Weak silt influences deeper soils.
Figure 6B	NaHCO_3_-Pi (pH, NLP, LP, Precipitation)	pH positively regulates NLP; NaHCO_3_-Pi positively affects NLP and LP; precipitation positively affects NLP.	pH→NLP (*p* < 0.01). NaHCO_3_-Pi→NLP (*p* < 0.01). NaHCO_3_-Pi→LP (*p* < 0.01). Precipitation→NLP (*p* < 0.05).	Precipitation drives NLP accumulation in deeper soil layers.
Figure 6C	NaOH-Po (pH, NLP, Precipitation, Temperature)	pH positively regulates NLP, while its effect on NaOH-Po is negative; NaOH-Po positively affects NLP; precipitation positively affects NLP, while temperature negatively affects NLP.	pH→NLP (*p* < 0.01). pH←NaOH-Po (*p* < 0.05). NaOH-Po→NLP (*p* < 0.01). Precipitation→NLP (*p* < 0.05). Temperature←NLP (*p* < 0.01).	Precipitation drives NLP accumulation, whereas temperature reduces NLP accumulation in deeper soil layers.
Figure 6D	HCl-Pi (pH, NLP, LP)	pH negatively regulates NLP, while its effect on HCl-Pi is positive; HCl-Pi positively affects NLP; NLP positively affects LP.	pH←NLP (*p* < 0.05). pH→HCl-Pi (*p* < 0.01). HCl-Pi→NLP (*p* < 0.01). NLP→LP (*p* < 0.01).	In the SEM model depicting the dynamics of HCl-Pi in deeper soil layers, the regulation of LP by NLP is the most significant.
Figure 8A	NaHCO_3_-Po (pH, NLP, LP, P and N Accumulation, SOC)	pH positively regulates NLP, while its effect on NaHCO_3_-Po is negative; NaHCO_3_-Po positively affects NLP and LP; NLP and LP positively affects P and N accumulation; N accumulation positively affects SOC.	pH→NLP (*p* < 0.01). pH←NaHCO_3_-Po (*p* < 0.01). NaHCO_3_-Po→NLP and LP (*p* < 0.01). NLP and LP→P and N Accumulation (*p* < 0.01). N Accumulation→SOC (*p* < 0.01).	Depth reduces phosphorus availability in forest soils. SOC played a central role in N accumulation but did not directly influence P accumulation in forest ecosystems.
Figure 8B	NaOH-Po (pH, NLP, LP, P and N Accumulation, SOC)	pH positively regulates NLP, while its effect on NaOH-Po is negative; NaOH-Po positively affects NLP; NLP and LP positively affect P and N accumulation; N accumulation positively affects SOC; NLP positively affects LP.	pH→NLP (*p* < 0.01). pH←NaOH-Po (*p* < 0.01). NaOH-Po→NLP (*p* < 0.01). NLP and LP→P and N Accumulation (*p* < 0.01). N Accumulation→SOC (*p* < 0.01). NLP→LP (*p* < 0.05).	Climatic factors influence N and P accumulation in forest soils.
Figure 8C	NaHCO_3_-Po (pH, NLP, LP, N Accumulation)	pH positively regulates NLP, while its effect on NaHCO_3_-Po is negative; NaHCO_3_-Po positively affects NLP, LP, and N accumulation; NLP positively affects LP.	pH→NLP (*p* < 0.01). pH←NaHCO_3_-Po (*p* < 0.01). NaHCO_3_-Po→NLP, LP and N Accumulation (*p* < 0.01). NLP→LP (*p* < 0.01).	Unlike forest soil, temperature and precipitation had weaker and non-significant effects on NLP, LP, and N accumulation in grasslands soil.
Figure 8D	NaOH-Po (pH, NLP, LP, N Accumulation)	pH positively regulates NLP, while its effect on NaOH-Po and LP is negative; NaOH-Po positively affects NLP and N accumulation; LP positively affects N accumulation; NLP positively affects LP.	pH→NLP (*p* < 0.01). pH←NaOH-Po and LP (*p* < 0.05). NaOH-Po→NLP and N Accumulation (*p* < 0.01). LP→N Accumulation (*p* < 0.01). NLP→LP (*p* < 0.01).	Grassland nutrient dynamics differ from forest biomes. LP exhibited a strong positive impact on N accumulation in the NaOH-Po model.
Figure 10A	Resin-P (pH, EC, NLP, N Accumulation, LP)	pH and EC positively regulate NLP, while their effect on Resin-P is negative; Resin-P negatively regulates N accumulation, while its effect on LP is positive.	pH and EC→NLP (*p* < 0.01). pH and EC←Resin-P (*p* < 0.01). Resin-P←N Accumulation (*p* < 0.01). Resin-P→LP (*p* < 0.01).	Resin-P is suppressed by both pH and EC in upper Japanese soils.
Figure 10B	NaOH-Pi (pH, EC, NLP, LP, P and N Accumulation)	pH and EC positively regulate NLP and NaOH-Pi, while pH negatively regulates LP; NaOH-Pi positively affects NLP, LP, P, and N accumulation; NLP negatively affects LP.	pH and EC→NLP and NaOH-Pi (*p* < 0.01). pH←LP (*p* < 0.01). NaOH-Pi→ NLP, LP, P and N Accumulation (*p* < 0.01). NLP←LP (*p* < 0.01).	pH enhances stable phosphorus forms.
Figure 10C	NaOH-Po (pH, LP, NLP, EC, P and N Accumulation)	pH negatively regulates LP and NLP, EC negatively regulates P accumulation, while pH and EC positively regulate NaOH-Po; NaOH-Po positively affects NLP and N accumulation, while its effect on LP is negative; NLP positively affects LP.	pH←LP (*p* < 0.05). pH←NLP (*p* < 0.01). EC←P Accumulation (*p* < 0.01). pH and EC→NaOH-Po (*p* < 0.01). NaOH-Po→NLP and N Accumulation (*p* < 0.01). NaOH-Po←LP (*p* < 0.01). NLP→LP (*p* < 0.01).	In the SEM model describing the dynamics of NaOH-Po in the 0–30 cm layer of Japanese blueberry soils, the regulation of LP by NLP exhibits the most significant effect.
Figure 10D	NaHCO_3_-Pi (pH, EC, NLP, LP, N Accumulation)	pH and EC positively regulate NLP, while their effect on NaHCO_3_-Pi is negative; pH negatively affects LP; NaHCO_3_-Pi positively affects NLP and N accumulation.	pH and EC→NLP (*p* < 0.01). pH and EC←NaHCO_3_-Pi (*p* < 0.01). pH←LP (*p* < 0.01). NaHCO_3_-Pi→LP and N Accumulation (*p* < 0.01).	Higher pH and EC reduce phosphorus mobility and enhance their stabilization in upper soil layers.
Figure 10E	NaHCO_3_-Po (pH, EC, NLP, LP, P Accumulation)	pH and EC positively regulate NLP and NaHCO_3_-Po, while their effect on LP is negative; NaHCO_3_-Po positively affects NLP and LP, while its effect on P accumulation is negative.	pH and EC→NLP and NaHCO_3_-Po (*p* < 0.01). pH and EC←LP (*p* < 0.01). NaHCO_3_-Po→NLP and LP (*p* < 0.01). NaHCO_3_-Po←P Accumulation (*p* < 0.01).	Higher pH and EC reduce phosphorus mobility and enhance their stabilization in upper soil layers.
Figure 10F	HCl-Pi (pH, EC, NLP, LP, P and N Accumulation)	pH and EC positively regulate NLP and HCl-Pi, while their effect on LP is negative; HCl-Pi positively affects NLP, while its effect on P and N accumulation is negative.	pH and EC→NLP and HCl-Pi (*p* < 0.01). pH and EC←LP (*p* < 0.01). HCl-Pi→NLP (*p* < 0.01). HCl-Pi←P and N Accumulation (*p* < 0.01).	HCl-Pi in the upper soil layers reduces both P accumulation and N accumulation.
Figure 10G	Residual-P (pH, EC, NLP, LP, N Accumulation)	pH and EC positively regulate NLP, while their effect on LP is negative; pH negatively affects Residual-P; EC positively affects Residual-P; Residual-P positively affects NLP, while its effect on N accumulation is negative.	pH and EC→NLP (*p* < 0.01). pH and EC←LP (*p* < 0.01). pH←Residual-P (*p* < 0.05). EC→Residual-P (*p* < 0.05). Residual-P→NLP (*p* < 0.01). Residual-P←N Accumulation (*p* < 0.01).	In the upper soil layers, pH inhibits Residual-P, while EC promotes its accumulation.
Figure 11A	Resin-P (pH, EC, NLP, LP)	pH and EC positively regulate NLP, while their effect on Resin-P is negative; EC negatively affects LP; Resin-P positively affects LP; NLP positively affects LP.	pH and EC→NLP (*p* < 0.01). pH and EC←Resin-P (*p* < 0.01). EC←LP (*p* < 0.05). Resin-P→LP (*p* < 0.01). NLP→LP (*p* < 0.01).	In the SEM model describing the dynamics of Resin-P in the 30–60 cm soil layer of Japanese blueberry fields, the regulation of LP by NLP shows a statistically significant effect.
Figure 11B	NaOH-Pi (pH, EC, NLP, LP, P Accumulation)	pH and EC positively regulate NLP, while their effect on LP is negative; EC positively regulates NaOH-Pi; NaOH-Pi positively affects NLP, LP, and P accumulation.	pH and EC→NLP (*p* < 0.01). pH and EC←LP (*p* < 0.01). EC→NaOH-Pi (*p* < 0.01). NaOH-Pi→NLP, LP and P Accumulation (*p* < 0.01).	EC promotes the stabilization of phosphorus in deeper soil layers of Japanese blueberry fields.
Figure 11C	NaOH-Po (pH, EC, NLP, N Accumulation, LP)	pH and EC positively regulate NaOH-Po; EC negatively affects LP; pH negatively affects NLP; NaOH-Po positively regulates NLP and N accumulation, while its effect on LP is negative; NLP positively affects LP.	pH and EC→NaOH-Po (*p* < 0.01). EC←LP (*p* < 0.01). pH←NLP (*p* < 0.01). NaOH-Po→NLP (*p* < 0.01). NaOH-Po→N Accumulation (*p* < 0.05). NaOH-Po←LP (*p* < 0.01). NLP→LP (*p* < 0.01).	In the SEM model describing the dynamics of NaOH-Po in the 30–60 cm layer of Japanese blueberry soils, the regulation of LP by NLP exhibits the most significant effect.
Figure 11D	NaHCO_3_-Pi (pH, EC, NLP, LP)	pH and EC positively regulate NLP, while their effect on NaHCO_3_-Pi is negative; NaHCO_3_-Pi positively affects LP and NLP.	pH and EC→NLP (*p* < 0.01). pH and EC←NaHCO_3_-Pi (*p* < 0.01). NaHCO_3_-Pi→LP and NLP (*p* < 0.01).	Inorganic phosphorus, such as NaHCO_3_-Pi, NaOH-Pi and HCl-Pi play a critical role in supporting P cycling through its influence on LP and NLP.
Figure 11E	NaHCO_3_-Po (pH, EC, LP, NLP, N Accumulation)	pH and EC positively regulate NaHCO_3_-Po, while their effect on LP is negative; EC positively regulates NLP; NaHCO_3_-Po positively regulates NLP, while its effect on N accumulation is negative; NLP positively affects LP.	pH and EC→NaHCO_3_-Po (*p* < 0.01). pH and EC←LP (*p* < 0.01). EC→NLP (*p* < 0.01). NaHCO_3_-Po→NLP (*p* < 0.01). NaHCO_3_-Po←N Accumulation (*p* < 0.05). NLP→LP (*p* < 0.05).	In the SEM model describing the dynamics of NaHCO_3_-Po in the 30–60 cm soil layer of Japanese blueberry fields, the regulation of LP by NLP shows a statistically significant effect.
Figure 11F	HCl-Pi (pH, EC, LP, NLP, N Accumulation)	pH and EC positively regulate HCl-Pi, while their effect on LP is negative; EC positively regulates NLP; HCl-Pi positively regulates NLP and LP, while its effect on N accumulation is negative; NLP negatively affects LP.	pH and EC→HCl-Pi (*p* < 0.01). pH and EC←LP (*p* < 0.01). EC→NLP (*p* < 0.01). HCl-Pi→NLP and LP (*p* < 0.01). HCl-Pi←N Accumulation (*p* < 0.01). NLP←LP (*p* < 0.01).	In the SEM model describing the dynamics of HCl-Pi in the 30–60 cm layer of Japanese blueberry soils, NLP exhibits a significant negative effect on LP.
Figure 11G	Residual-P (pH, EC, LP, NLP, P Accumulation)	pH and EC negatively regulate LP, while pH positively affects NLP; pH negatively affects Residual-P, while EC positively affects Residual-P; Residual-P positively affects NLP, while its effect on P accumulation is negative; NLP positively affects LP.	pH and EC←LP (*p* < 0.01). pH→NLP (*p* < 0.01). pH←Residual-P (*p* < 0.01). EC→Residual-P (*p* < 0.01). Residual-P→NLP (*p* < 0.01). Residual-P←P Accumulation (*p* < 0.05). NLP→LP (*p* < 0.01).	In the SEM model describing the dynamics of Residual-P in the 30–60 cm soil layer of Japanese blueberry fields, the regulation of LP by NLP shows a statistically significant effect.
Figure 13A	NaHCO_3_-Po (pH, Depth, NLP, LP, P and N Accumulation, Temperature)	pH and depth negatively regulate NaHCO_3_-Po, while NaHCO_3_-Po positively affects NLP and LP; NLP and LP positively affect P and N accumulation; temperature positively affects N accumulation; NLP positively affects LP.	pH←NaHCO_3_-Po (*p* < 0.05). Depth←NaHCO_3_-Po (*p* < 0.01). NaHCO_3_-Po→NLP and LP (*p* < 0.01). NLP and LP→P and N Accumulation (*p* < 0.01). Temperature→N Accumulation (*p* < 0.01). NLP→LP (*p* < 0.01).	In the SEM model describing the dynamics of NaHCO_3_-Po in the symbiotic microbial activity layer, the regulation of LP by NLP demonstrates a statistically significant effect, and temperature exerts a positive influence on N accumulation.
Figure 13B	NaHCO_3_-Po (pH, Depth, Silt, NLP, LP, P and N Accumulation)	pH and depth negatively regulate NaHCO_3_-Po, while silt positively regulates NaHCO_3_-Po; pH positively affects NLP; NaHCO_3_-Po positively affects NLP and LP; LP positively affects P and N accumulation; NLP positively affects P accumulation; NLP positively affects LP.	pH and depth←NaHCO_3_-Po (*p* < 0.01). Silt→NaHCO_3_-Po (*p* < 0.05). pH→NLP (*p* < 0.05). NaHCO_3_-Po→NLP and LP (*p* < 0.01). LP→P and N Accumulation (*p* < 0.01). NLP→P Accumulation (*p* < 0.01). NLP→LP (*p* < 0.05).	In the SEM model describing the dynamics of NaHCO_3_-Po in the inactive symbiotic microbial layer, the regulation of LP by NLP demonstrates a statistically significant effect.
Figure 13C	NaOH-Po (pH, LP, NLP, P and N Accumulation)	pH negatively regulates NaOH-Po and LP, while its effect on NLP is positive; NaOH-Po positively affects NLP; LP positively affects P and N accumulation; NLP positively affects P accumulation; NLP positively affects LP.	pH←NaOH-Po and LP (*p* < 0.05). pH→NLP (*p* < 0.01). NaOH-Po→NLP (*p* < 0.01). LP→P and N Accumulation (*p* < 0.01). NLP→P Accumulation (*p* < 0.01). NLP→LP (*p* < 0.01).	In the SEM model describing the dynamics of NaOH-Po in the inactive symbiotic microbial layers, the regulation of LP by NLP is more pronounced compared to that in the dynamics of NaHCO_3_-Po.

Note: The arrow → indicates positive regulation, while the arrow ← indicates negative regulation.

## Data Availability

The data are included in the article and [OSF] [https://osf.io/xt84q/, accessed on 3 December 2024].

## Supplementary Materials

The following supporting information can be downloaded at: https://www.mdpi.com/article/10.3390/plants14020189/s1, File S1: global raw data. File S2: global training data. File S3: global predicted data.
